# Examination of Electrolyte Replacements in the ICU Utilizing MIMIC-III Dataset Demonstrates Redundant Replacement Patterns

**DOI:** 10.3390/healthcare9101373

**Published:** 2021-10-14

**Authors:** Mousa Ghannam, Parasteh Malihi, Krzysztof Laudanski

**Affiliations:** 1 School of Dental Medicine, Leonard Davis Institute of Healthcare Economics, University of Pennsylvania, Philadelphia, PA 19104, USA; mousag@upenn.com (M.G.); pmalihi@upenn.com (P.M.); 2 Department of Anesthesiology and Critical Care, Department of Neurology, Leonard Davis Institute of Healthcare Economics, University of Pennsylvania, Philadelphia, PA 19104, USA

**Keywords:** electrolyte repletion, decision making, ICU, MIMICS

## Abstract

Electrolyte repletion in the ICU is one of the most ubiquitous tasks in critical care, involving significant resources while having an unclear risk/benefit ratio. Prior data indicate most replacements are administered while electrolytes are within or above reference ranges with little effect on serum post-replacement levels and potential harm. ICU electrolyte replacement patterns were analyzed using the MIMIC-III database to determine the threshold governing replacement decisions and their efficiency. The data of serum values for potassium, magnesium, and phosphate before and after repletion events were evaluated. Thresholds for when repletion was administered and temporal patterns in the repletion behaviors of ICU healthcare providers were identified. Most electrolyte replacements happened when levels were below or within reference ranges. Of the lab orders placed, a minuscule number of them were followed by repletion. Electrolyte repletion resulted in negligible (phosphate), small (potassium), and modest (magnesium) post-replacement changes in electrolyte serum levels. The repletion pattern followed hospital routine work and was anchored around shift changes. A subset of providers conducting over-repletion in the absence of clinical indication was also identified. This pattern of behavior found in this study supports previous studies and may allude to a universal pattern of over-repletion in the ICU setting.

## 1. Background

Electrolyte repletion is a ubiquitous routine intervention carried out to maintain a homeostatic range of compounds in serum [1,2]. An abnormal level of most abundant electrolytes (sodium, chloride, potassium, magnesium, phosphate, and calcium) is linked to heart, coagulation, muscle, and nervous system pathologies, as these electrolytes are critical for electrical signal conduction and enzyme activity [3,4,5,6,7]. 

As with any other therapeutic maneuver, the goal of electrolyte repletion is to improve outcomes while reducing harm [8,9]. The decision making is based in very significant way on a comparison between electrolyte levels and reference ranges, which are considered physiological [1,2,10]. These references ranges are not delineated but rather fuzzy, as there is only some evidentiary support that relative hyperkalemia (potassium level between 4 and 4.5 mEq/dL) can prevent atrial fibrillation after cardiac surgery, but this is not a uniform finding [3,4,11,12,13]. Relative hypermagnesemia can stabilize the heart’s electrical activity, terminate atrial fibrillation, and decrease seizure likehood while relieving bronchospasms in certain conditions when a drug is given as bolus resulting in a rapid increase in electrolytes with normalization afterward [1,5,6,13]. Consequently, medical professionals may take the laboratory value of electrolyte levels in serum in context or subject it to their interpretation. A lack of consensus regarding what the optimal electrolyte levels are leads to high replacement heterogeneity [10,14,15]. However, attaining optimal levels is difficult, as electrolyte levels are affected by several physiological parameters: other electrolyte levels, drugs, and ongoing illness processes [1,6,13]. There is also no direct proof of how much these important clinical variables weigh into clinical decision processes. Moreover, several assumptions about the beneficial effect of electrolyte replacement may be incorrect [16,17,18]. The effect of interventions is often overestimated by providers [12,19]. Providers tend to emphasize that the depletion of electrolytes occurs more often than high levels of electrolytes [10,20,21]. Intensive repletion efforts may harm patients when replacements are given to patients with an already high electrolyte level [19]. Repletion regimens vary by institution, individual patient factors, and provider factors, resulting in clinical treatment following hospital routine and not the patients’ needs [15]. This may result in the delivery of interrupted care during the day due to work organization and non-clinical indicators [22,23,24]. The local culture in electrolyte replacement may be another factor influencing decision making for electrolyte replacement. Even when standardized protocols are implemented to improve patient outcomes, adherence is scattered [10,20,25,26,27,28,29]. Patterns of decision making overlay this complex network of factors, affecting electrolytes in healthcare in general [7,15,20,30]. The intensive care unit environment exaggerates all these factors while adding the time pressure of a highly dynamic and cognitively challenging environment [7,9,15,30,31,32,33]. Simultaneously, medical training struggles to adequately equip medical professionals with the skills to execute decisions rooted in evidence and based on heuristics [9,34,35,36]. The complex network of variables determines a physician’s decision of whether to replace electrolytes and the only successful attempts have utilized complex statistical methods and artificial intelligence tools.

Current patterns and the available literature suggest superfluous electrolyte repletion patterns in the ICU [30]. There is also a gap in knowledge in understanding how electrolyte lab values are analyzed and considered when electrolytes are replaced or not. Furthermore, a prior study did not explore the nature of electrolyte replacement thresholds (discrete or continuous), did not look into scenarios where electrolytes were not repleted, and was subjected to bias, as the study came from one institution [30]. Extending the research project to MIMIC-III allows for cross-validation of the results of the prior data, especially in terms of efficiency of the electrolyte replacements [30]. Analyzing pre-existing lab values may lead to a better understanding as to whether providers consider a discrete value to make the decision to replace or, rather, utilize a range of values. Considering that providers place different weights on too low vs. too high electrolyte levels, such a threshold may be different, but it is unknown if it is the same in repletion vs. no-repletion clinical scenarios [10,20,31]. It is also unclear if these patterns of electrolyte replacements are influenced by a pre-existing condition, patient’s characteristics, or the unit assigned. 

## 2. Materials and Methods

### 2.1. Sample 

The data set used in this study was from the MIMIC-III critical care database [37]. This database comprises de-identified health records for over forty thousand critical care patients admitted to the ICU in the Beth Israel Deaconess Medical Center (Boston, MA, USA) between 2001 and 2012. In addition, the database includes demographics, vital signs, laboratory test results, medication, and caregiver information for 2008–2012. The dataset was assembled into a relational database (PostgreSQL) and queried as necessary, and a statistical analysis was conducted using R-packages software [38,39].

**Figure 1:**
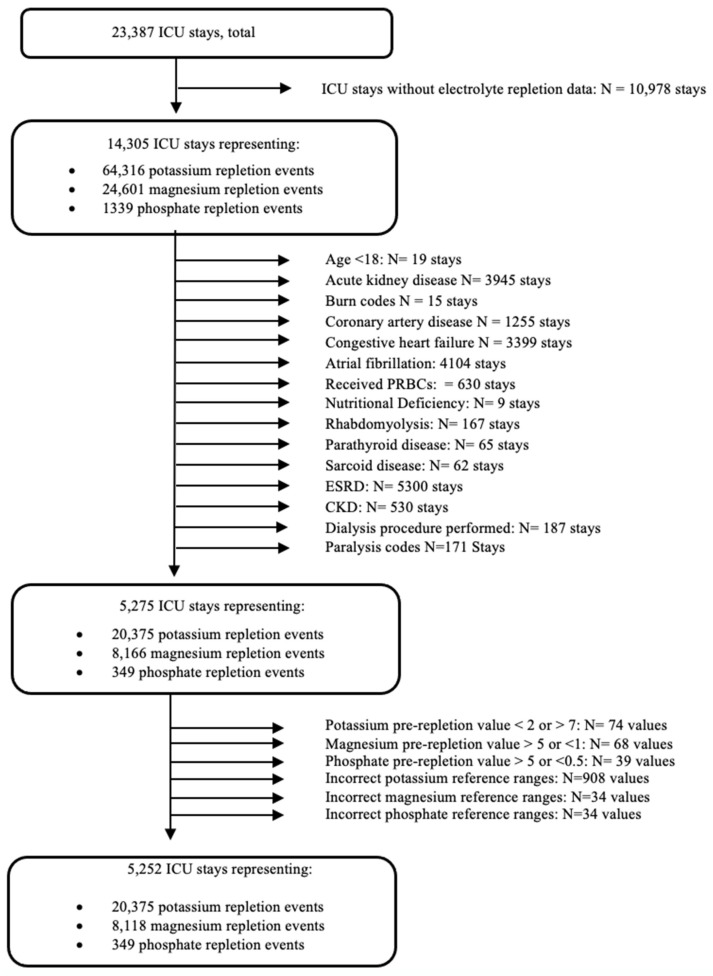
The structure of the data analysis.

The MIMIC-III database, limited to Metaview data, includes critical care entries containing information on 23,387 ICU stays. The screening process began by excluding ICU stays lacking electrolyte repletion or a lab value (*n* = 10,978) (Figure 1). Then, similar data exclusion and inclusion criteria were used as in the prior study to minimize heterogeneity using the International Classification of Diseases (ICD-9) codes (Supplementary Table S1) [19]. In this study, patients under 18 years old (*n* = 19) were excluded. The MIMIC-III database contains a list of diagnoses in order of relevancy for each patient, but only the first diagnosis was selected as the object of data analysis [37]. Exclusions included conditions associated with several electrolyte disturbances complicating the provider’s decision-making process, resulting in a very complex and potentially unreliable analysis [1,2,19]. 

Consequently, events involving the transfusion of packed red blood cells (*n* = 630), rhabdomyolysis (*n* = 1167), a parathyroid disease of any kind (*n* = 65), sarcoid disease (*n* = 62), chronic kidney disease (*n* = 530), acute kidney disease (*n* = 33,945), and end-stage renal disease (*n* = 5300) were eliminated. Additionally, encounters where the patient had coronary artery disease (*n* = 1255), congestive heart failure (*n* = 3399), atrial fibrillation (*n* = 4104), nutritional deficiency (*n* = 9), and paralysis (*n* = 171) were excluded from further analysis. Additionally, events where a dialysis procedure was performed were also excluded (*n* = 187). From the remaining 5275 ICU stays, potassium pre-repletion values below 2 or above 7 (*n* = 74), magnesium pre-repletion values greater than 5 or less than 1 (*n* = 68), and phosphate pre-repletion values below 0.5 and above 5 (*n* = 39) were filtered out as errors.

The nominal reference values in the MIMICS-III database are 3.7–5.7 for potassium, 2.5–4.5 for magnesium, and 2.5–4.5 for phosphate, with values outside of these flagged as abnormal.

### 2.2. Study Workflow 

**Figure 2:**
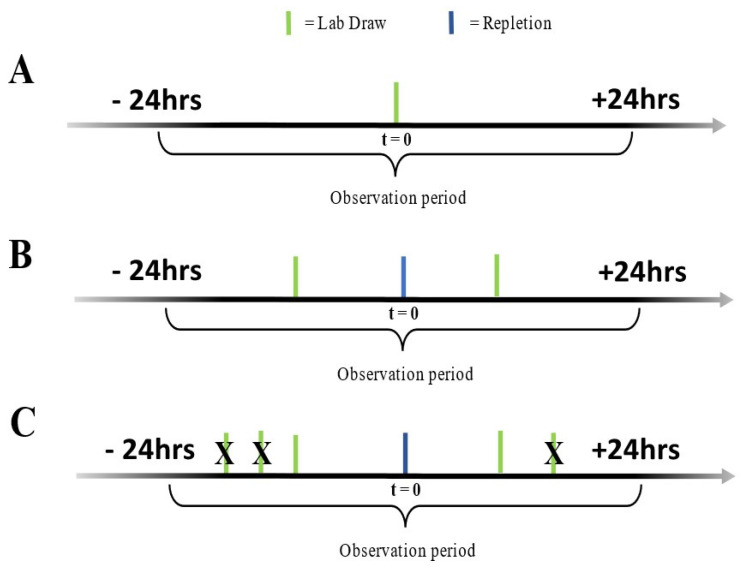
Three different clinical scenarios around the electrolyte replacements. In the first case, only electrolyte lab draw was obtained without preceding electrolyte replacement, and subsequent supplementation was given within 24 h (**A**). The lab draw was preceded by a singular electrolyte replacement (**B**) or multiple electrolyte replacements (**C**) in two other scenarios.

In this process, the timeframe for analysis followed a 24-h window from the time when the lab value was drawn. This approach resulted in three different scenarios. In the first instance, a lab value was drawn, but there was no instance of electrolyte replacement after 24 h (***L only/No repletion*** (Figure 2A). If a singular lab value was followed by a repletion, it was assigned to the second scenario (***1L → 1R***) (Figure 2B). These cases constituted 51.1% of all cases where repletion took place. Finally, in cases where multiple lab values preceded electrolyte replacement in 24-h windows, the closest value was used, and the others were ignored (Figure 2C). This was called a multiple lab scenario (***ML → 1R***), and the frequency of these events was 48.9%. Subsequent electrolyte lab values were searched for within a 24-h window, and the lab value closest to the order placement in all three scenarios were considered. This workflow is a reasonable reflection of routine hospital performance [8,40,41,42].

### 2.3. Statistical Analysis 

The Shapiro–Wilk W test and distribution plots were used to test the normality of distribution variables. The parametric variables are expressed as mean ± SD and compared using *t*-Student. The effect size was estimated as *d*-Cohen statistics. Regression analysis was conducted by addition, and a subtraction technique with the ordinary least squares’ method was used for all linear regressions. Correlations were reported as Pearson *r* values. A double-sided *p*-value less than 0.05 was considered statistically significant for all tests. Statistical analyses were performed with specified R packages [7,38,39,42,43,44,45,46,47].

## 3. Results

### 3.1. Relationship between Ordering Serum Electrolyte Replacements and Their Threshold for Replacements

**Figure 3:**
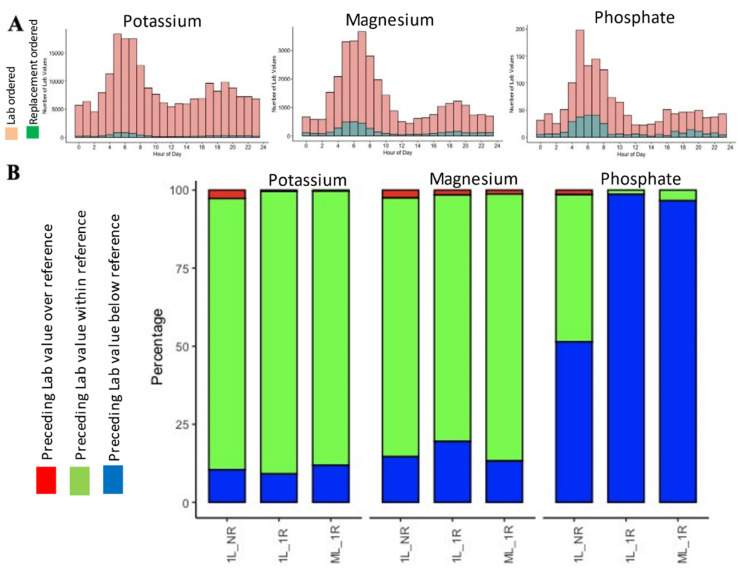
Only a minority of the lab values resulted in clinical intervention (**A**). In the case of potassium and magnesium repletion, trigger values were within the normal limit but not in the case of hypophosphatemia, which was a more frequent trigger for replacement than the normal phosphate level (**B**).

The blood collection and lab reporting followed certain time patterns with only 3.24% of potassium, 10.37% magnesium, and 16.62% of phosphate lab orders resulting in repletion (Figure 3A). Overwhelmingly, orders for the replacements of potassium and magnesium were placed when serum levels were in the nominal ranges for every scenario analyzed here. In contrast, phosphate was replaced more often when lab values indicated hypophosphatemia (Figure 3B; Table 1). Irrespective of the clinical scenario discussed in this manuscript, the frequencies of the electrolytes below, within, or over the nominal value were similar (Figure 3B). 

**Figure 4:**
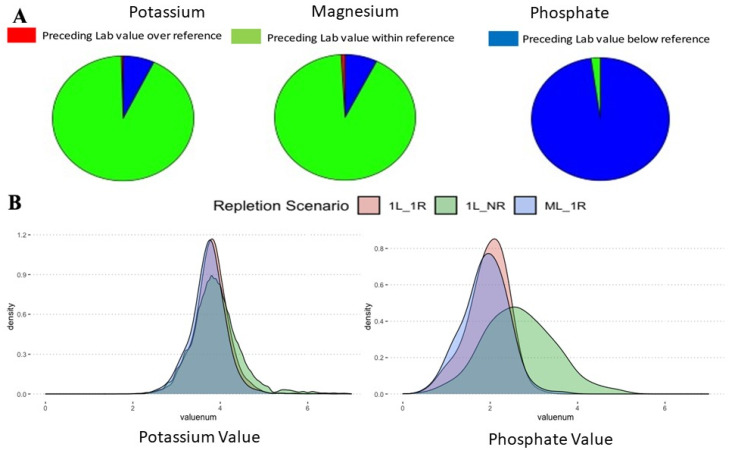
In the case of lab values triggering clinical intervention, most cases for potassium and magnesium have a nominal level of electrolyte but not phosphate (**A**). Concomitantly, the threshold for repletion was very fuzzy and overlapped in the case of potassium and phosphate when repletion did take place (1L_1R) vs cases when repletion did not happen (1L_NR) (**B**).

When considering instances where repletion took place, the preceding lab value of the electrolytes was mostly within the normal value for potassium and magnesium but not for phosphate (Figure 4A; Table S1). When the instances where electrolytes were not replaced (1L → NR) versus when they were replaced (1L → 1R and ML → 1R) were compared, the preceding electrolyte level was different. With respect to effect size, repletion mattered little for both potassium and magnesium (Table S1). At the same time, phosphate had many more cases where providers decided not to replace at nominal and above reference levels (Table S1). The top ten diagnoses for each electrolyte repletion falling above and below thresholds were distinct for each electrolyte (Supplementary Figure S1; Supplementary Table S3).

### 3.2. Investigating Misses and Near Misses among Electrolyte Replacement Patterns 

The analysis revealed instances when preceding electrolyte levels were above the limit. The frequency of these instances was 0.38% for potassium, 1.4% for magnesium, and 0% for phosphate (Table S1). The relative scarcity of these cases precluded any meaningful analysis, but their clinical characteristic is heterogeneous (Supplementary Table S2; Supplementary Figure S1).

### 3.3. The Workflow and Triggers in Case of Electrolyte Replacements

**Figure 5:**
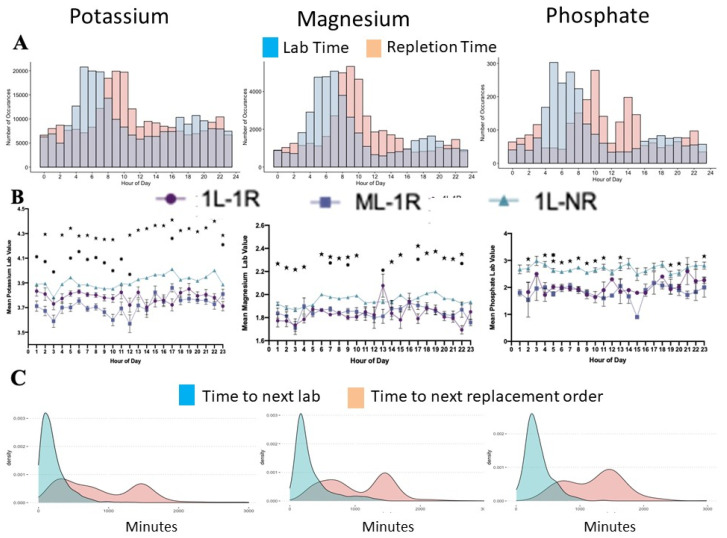
When the electrolyte order was placed, the lab value and replacement seemed to follow hospital routine (**A**). However, the threshold did not fluctuate considerably over 24 h, and the most significant difference in the threshold was when multiple lab values preceded the electrolyte replacement order (**B**). The follow-up lab values were executed in bimodal distribution (**C**), but the peaks depended on electrolyte type.

Most of the electrolyte lab values were taken around 6 a.m., followed by repletion at 9 a.m. in cases where an electrolyte was replaced (Figure 5A). On average, the replacement was ordered within 217.20 min (3 h 36 min) for potassium, 323.29 min (5 h 18 min) for magnesium, and 339.83 min (5 h 36 min) for phosphate for the charting of the initial lab values. A follow-up lab value was ordered on average within 443.45 min (7 h 18 min), 643.10 min (10 h 42 min), and 567.13 min (9 h 24 min) for potassium, magnesium, and phosphate, respectively (Figure 5B).

The threshold of electrolyte replacement remained steady for 24 h but varied between 1L->1R and 1L->MR versus 1L->NR (Figure 5B). In addition, if multiple electrolyte lab values were requested, a different bimodal distribution of lab orders was seen (Figure 5C).

### 3.4. The Effectiveness of Electrolyte Replacement Results in Modest Changes in Post-Repletion Potassium

**Figure 6:**
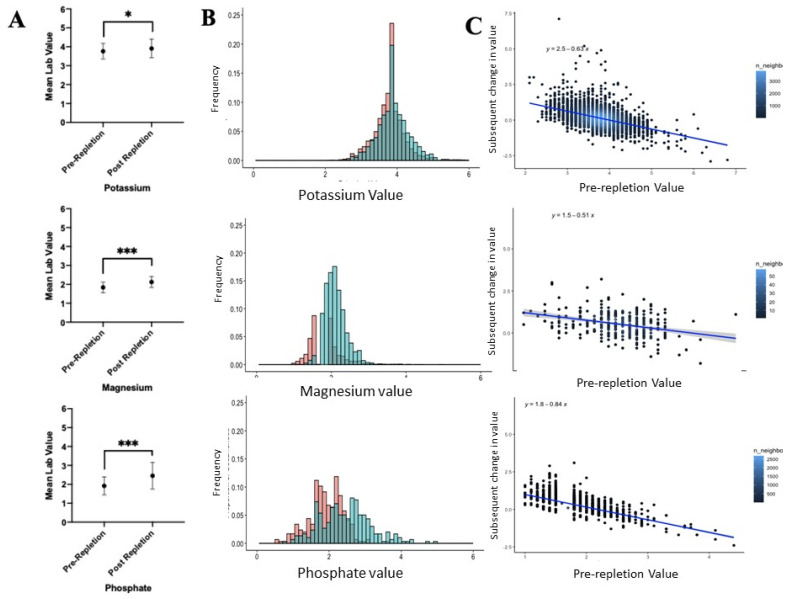
Electrolyte replacement resulted in a modest increase in potassium, while changes in magnesium and phosphate were much more pronounced when pre-post value were analyzed as averages (**A**) or as distribution of values. (**B**). For all of the electrolytes, the most significant variable determining post-repletion level was the preceding hypo-electrolyte state (**C**). * Refers to the effect size of the d-Cohen statistic, with * and *** corresponding to small, moderate, and large, respectively.

Orders to replace electrolytes resulted in a highly significant difference before and after repletion, but the effect size of repletion varied by electrolyte and, at best, should be judged as moderate as demonstrated by the *d*-Cohen values. Potassium demonstrated a small effect size of 0.32, while magnesium and phosphate resulted in large effect sizes of 1.08 and 0.87, respectively (Figure 6A,B).

Multiple regression analysis determined several factors that correlated with changes in the potassium level (Table 2). The primary predictor of a significant increase in electrolyte levels was pre-repletion levels (Figure 5C). However, analysis of several other factors and their contribution had a significantly less impactful effect, as demonstrated by the regression coefficient (Table 2).

### 3.5. The Effect of Clinical Co-Variables and Patient Location on Electrolyte Thresholds

**Figure 7:**
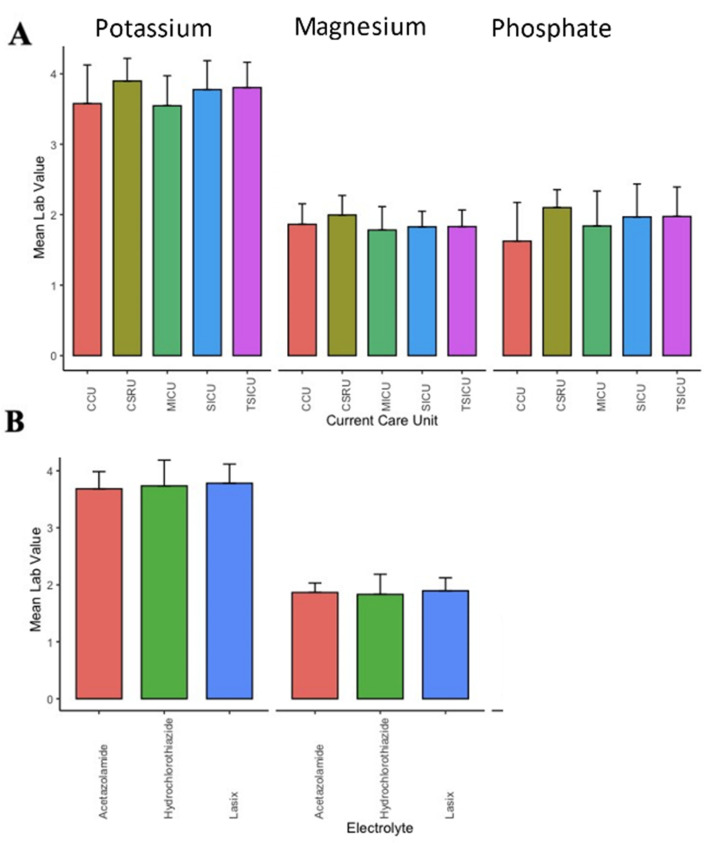
Significant differences in the threshold for replacement exist between different ICUs for potassium and magnesium but not phosphate (**A**). Being on a potassium-wasting diuretic (furosemide) or a potassium-sparring diuretic (acetazolamide) did not result in alteration of the threshold to replace potassium and magnesium (**B**).

Finally, the heterogeneity of the threshold for order replacements was examined in the context of the patients’ locations and medications, which had a profound effect on electrolyte levels. The thresholds to replace were identified as being different when the ICU type was considered for potassium (F(4;6691) = 162.056; *p* < 0.0001) and magnesium (F(4;3472) = 52.41; *p* < 0.0001) but not phosphate (F(4;282) = 1.96; *p* = ns) (Figure 7A). Being on medications commonly associated with electrolyte abnormalities did not result in a statistically significant effect on the threshold for serum electrolyte replacement (Figure 7B).

## 4. Discussion

This study demonstrated that a significant number of orders measuring the serum level of electrolytes resulted in no subsequent clinical interventions. The practice of electrolyte repletion followed hospital workflow. When healthcare providers placed an order for electrolyte replacement, the pre-existing lab value was, overwhelmingly, within normative values. This behavior is consistent with a prior study where a different data set was used [19]. However, significantly fewer instances where electrolytes were replaced when the pre-existing value was above nominal were identified [19]. This may suggest a different safety or practice policy. Irrespective of the reason, this demonstrates a difference in iatrogenic risk between the two institutions. The average serum value-producing repletion remained constant throughout the day, but the average lab value that prompted non-repletion fluctuated over 24 h. The effects of electrolyte repletion on the post-repletion levels of electrolytes were small. The threshold for electrolyte replacement varied depending on the unit but not the concomitant utilization of medications interfering with potassium homeostasis that also had some diurnal variability. In general, the data showed significant waste due to over-ordering lab values and ineffective repletion patterns.

Investigating electrolyte repletion in the MIMICS ICU database revealed over-repletion patterns by providers from a major hospital system in the Boston area. Similarities in physician behavioral patterns in other institutions support the presence of over-repletion patterns in the ICU. This may allude to a universal issue of over-repletion secondary to the perception that less risk is in inaction vs. initiation of treatment [15,19,21]. This bias may be particularly strong in units where electrolyte and cardiac abnormalities are of particular concern (HVICU and CICU), even though evidence has demonstrated the ineffectiveness of pre-emptive repletion of electrolytes in general [1,2,4,12,18,29]. However, in this study, cardiac units did not demonstrate a lower threshold for electrolyte replacement. Conversely, the threshold for potassium replacement was the highest, which suggests that a higher goal is set with providers attempting to use even higher levels. Moreover, the diagnoses related to under-repletion were mostly non-cardiac, which is counterintuitive to the standard practice of being more aggressive with potassium replacement in the cardiac population. Furthermore, there was a weak correlation between electrolyte lab values and the propensity of the provider to replace electrolytes. The relation was linear and fuzzy instead of being a discrete value as evidence-based data would suggest. This study demonstrated that healthcare providers decide to replace or not replace electrolytes at a similar threshold, suggesting that other factors play a role. The regression analysis demonstrated that most of the factors listed by the literature as a critical determinant of electrolyte replacement do not contribute significantly to the decision as to whether to replace or not. Consequently, the factors that are likely to drive the electrolyte replacement process is the hospital culture instead of well-established evidence [1,10,20,21,28,34]. The fact that shift changes seemed to correlate with repletion is a strong argument for this. Significant common motifs in making these decisions judging from the variables incorporated into the analysis could not be found, including a potassium-wasting or -sparring diuretic. The influence of several other variables was small, even though their potential role was identified in alternative studies. This does not come as a surprise, as the medical decision-making process is often rooted in several variables, and the intricacies are often not accessible to the decision-making individual. Cultural inertia is often linked to the continuation of medical treatment, even if no evidence of support is present [48,49].

This study also analyzed the time dynamics of electrolyte replacement and found many electrolyte repletion orders for potassium, phosphate, and magnesium at around 7 a.m. (major peak) and then again at 6 p.m. (minor peak). Interestingly, this “circadian rhythm” pattern was found in a similar study analyzing provider behavior in another hospital system [19]. These major and minor peaks coincide with provider shift changes more than patient factors and may allude to a more informal repletion pattern. While patient outcomes and adverse events were not analyzed in this study, the hypothesis remains that this informal pattern of electrolyte repletion does not decrease the incidence of adverse events. One reason for the low effectiveness of potassium and magnesium replacement may be related to a large gap in care. Replacing potassium takes a significant amount of time. Furthermore, since potassium is an intracellular cation, most potassium will be translocated beyond serum [1,2]. Consequently, electrolyte replacement should be more frequent and potentially at higher doses to achieve a meaningful change in serum. This would suggest that more aggressive techniques of replacement should be utilized, especially in the case of critically ill patients. One approach to improvement is the development of a protocolized algorithm, but most studies have demonstrated increased compliance and an improved level of serum electrolytes despite a lack of evidence for a change in the clinical outcome [1,27,28,29]. This study demonstrates that electrolyte replacement ineffectiveness results from the providers’ behavior and healthcare organization tailored to patients’ needs and hospital workflow. That problem was identified before, but only a few studies could quantify the level of routine care [50,51]. Reduction is achievable using simple education or software solutions, yet the practice persists [52,53]. A similar problem in other testing modalities pointed toward an overall culture-driven mechanism instead of toward patient-centered care. A reduction in lab tests reduces error, improves quality of life, and accelerates healing [51].

Several limitations urge caution when analyzing the data. It is difficult to know when the healthcare providers were made aware of the results from the lab draws. Note that the times associated with these results are the time of fluid acquisition, not the time that the values were made available to the clinical staff. This may result in a situation where the lab is delayed beyond 24 h. However, this seems to be unlikely considering the performance of current lab reporting systems. Electrolyte replacement protocols from the hospital providing the data used in this study were unfortunately not available. Reference ranges dictated from the computer system were used as the guide to repletion. Consequently, there was assumed to be a lack of protocol, given that most of the protocolization of healthcare delivery happened in the last ten years. It was also assumed that typical printouts give the result and reference ranges, thus giving an immediate frame for the provider. As a result, it is not possible to assess whether the repletion orders made without specific indication were in line with existing protocols [10,15,21,30,34]. Deviation from these standard levels may be attributed to the disparity in optimal electrolyte levels across practices. Ascertaining the effect of provider type (physicians, advanced practice providers, etc.) was not possible, which may be a significant factor in electrolyte decision-making processes. Yet, the variability within provider behavior highlights the lack of compliance to a repletion protocol. Similarities to replacement patterns found in another hospital system and from a different time frame raise the question of whether existing protocols are a strong influencing factor in these behaviors. Alternatively, the patterns may result from non-compliance or the lack of standardized guidelines. In magnesium, electrolyte replacement is often driven not by the absolute level but by a clinical indication [2,13]. This can potentially introduce heterogeneity and is most likely reflected in some incidences of repletion of magnesium when the level was over the normative value.

This study suggests that a more effective process is needed in the field of electrolyte replacement. Currently, there is a significant over-ordering of lab values with little actionable decision. When decision to intervene is made, the decision does not seem to adhere to clinical evidence and is inconsistent. This study most likely confirmed the impression of all practicing physicians in the USA regarding over-ordering and unclear guidance in several “routine” tasks. Implementation of the protocol was suggested to improve the situation, but protocols are rigid, met occasionally with resistance, and cannot account for other variables. More effective changes may be made by focusing on a more targeted system of ordering from the lab, starting with abandoning scheduled lab draws and designing a demand- or clinical question-driven system. Regarding replacement prescription, utilizing mathematical modeling to predict optimal timing and dose is a potential solution.

## 5. Conclusions

Analysis of the MIMICS ICU database highlights a pattern of electrolyte repletion not in line with the needs of critical care patients in terms of workflow and effectiveness.

## Figures and Tables

**Table 1:** Comparison pre-order lab values for all three major electrolytes during the three clinical scenarios.

		Lab Interpretation	N	%	*p* and *d*-Cohen as Compared to a Threshold at the Repletion Reference Value	*p* and *d*-Cohen as Compared to a Threshold at the Nominal Reference Value
**Potassium**	Non-repletion	Above	5790	2.71	**6.05 ± 0.83 ^#^ [d = 0.52]**	≪0.00001; **3.05**
		Within	185,666	86.81	**3.95 ± 0.42 ^#^ [d = 0.30]**	Nominal
		Below	22,410	10.48	**3.00 ± 0.26 ^#^ [d = 0.08]**	≪0.00001, 2.94
	Repletion	Above	27	0.38	5.69 ± 0.42,	≪0.00001, 5.20
		Within	6400	89.31	3.84 ± 0.31	Nominal
		Below	739	10.31	3.02 ± 0.19	≪0.00001, 3.11
**Magnesium**	Non-repletion	Above	812	2.51	2.96 ± 0.70	≪0.00001, 3.32
		Within	26,823	82.88	**2.03 ± 0.22 ^#^ [d = 0.73]**	Nominal
		Below	4729	14.61	**1.38 ± 0.16 ^#^ [d = 0.24]**	≪0.00001, 3.4
	Repletion	Above	55	1.47	3.06 ± 0.39	≪0.00001, 3.64
		Within	3041	81.24	1.90 ± 0.16	Nominal
		Below	647	17.29	1.41 ± 0.12	≪0.00001, 3.74
**Phosphate**	Non-repletion	Above	22	1.49	4.85 ± 0.49	≪0.00001, 4.86
		Within	696	47.03	3.28 ± 0.34	Nominal
		Below	762	51.49	**2.05 ± 0.46 ^#^ [d = 0.37]**	≪0.00001, 2.76
	Repletion	Above	0	0	0	NA
		Within	7	2.37	3.04 ± 0.28	Nominal
		Below	288	97.63	1.89 ± 0.43	0.00002, 3.14

Denominates statistically significant difference instances for electrolytes when comparing three different lab interpretations within repletion vs. non-repletion for each given electrolyte with the nominal value as a reference and with d-Cohen statistic in parenthesis. ^#^ Denominates statistically significant difference instances for an electrolyte for one of the given scenarios when electrolyte was replaced vs. not replaced with d-Cohen statistic in parenthesis. Bolded number demonstrate statistically significant differences when repletion took place vs when it was not ordered.

**Table 2:** Regression analysis for the factors contributing to the decision to replace electrolytes.

		Potassium	Magnesium	Phosphate
**Patient Weight**	Coefficient (SD)	**0.001** *** (−0.0003)	0.0004 (−0.0004)	0.004 (−0.002)
Age	Coefficient (SD)	**−0.001** ** (−0.0003)	**−0.0004** * (−0.0003)	**0.002** * (−0.001)
Sodium	Coefficient (SD)	**−0.013** *** (−0.002)	−0.001 (−0.002)	**−0.026** ** (−0.01)
Anion Gap	Coefficient (SD)	**−0.004** * (−0.002)	**0.004** * (−0.002)	−0.011 (−0.012)
Creatine	Coefficient (SD)	−0.014 (−0.034)	**−0.078** ** (−0.032)	−0.196 (−0.181)
BUN	Coefficient (SD)	0.001 (−0.001)	0.001 (−0.001)	0.008 (−0.006)
WBC	Coefficient (SD)	0.001 (−0.001)	0.0001 (−0.001)	0.007 (−0.006)
Unit	Coefficient (SD)	**−0.002** *** (−0.0004)	**−0.001** *** (−0.0004)	0.0002 (−0.002)
Magnesium	Coefficient (SD)	0.018 (−0.019)	**0.070** *** (−0.026)	−0.074 (−0.052)
Phosphate	Coefficient (SD)	**0.025** *** (−0.008)	**−0.017** ** (−0.008)	0.043 (−0.059)
Chloride	Coefficient (SD)	**0.008** *** (−0.002)	0.001 (−0.002)	0.001 (−0.008)
Heart Rate	Coefficient (SD)	**−0.001** * (−0.0004)	**−0.001** *** (−0.0004)	**−0.004** ** (−0.002)
Respiratory Rate	Coefficient (SD)	**−0.004** *** (−0.001)	0.001 (−0.001)	−0.005 (−0.006)
Glucose	Coefficient (SD)	**−0.0002** ** (−0.0001)	0.0002 (−0.0001)	−0.001 (−0.001)
Systolic BP	Coefficient (SD)	−0.0002 (−0.0003)	−0.001 (−0.0004)	**−0.004** * (−0.002)
Potassium	Coefficient (SD)	**0.188** *** (−0.014)	**−0.032** ** (−0.015)	0.096 (−0.064)
Constant	Coefficient (SD)	**4.081** *** (−0.228)	**2.070** *** (−0.262)	**5.522** *** (−1.232)
Observations		2761	1520	165
R^2^		0.126	0.034	0.201
Adjusted R^2^		0.121	0.023	0.109
Residual Std. Error		0.392 (df = 2743)	0.295 (df = 1502)	0.443 (df = 147)
F Statistic		**23.322** *** (df = 17; 2743)	**3.090** *** (df = 17; 1502)	**2.175** *** (df = 17; 147)

* *p* < 0.1; ** *p* < 0.05; *** *p* < 0.01. Statistically significant differences bolded.

## Data Availability

Data are publicly available on the MIMICS website, while the R code is available on GitHub.

## Supplementary Materials

The following are available online at https://www.mdpi.com/article/10.3390/healthcare9101373/s1, Table S1. List of exclusion ICD-9 diagnoses and their codes. Table S2. Diagnoses and frequencies of the instances when over-repletion occurred. Table S3. Diagnoses and frequencies of the instances when under-repletion occurred. Figure S1. Top ten diagnoses when repletion was ordered when the preceding lab value was above or below the reference for potassium and magnesium.
