# Supplementary material for: Examination of Electrolyte Replacements in the ICU Utilizing MIMIC-III Dataset Demonstrates Redundant Replacement Patterns
Source: Healthcare (Basel). 2021 Oct 14;9(10):1373. doi: 10.3390/healthcare9101373 (PMC8536187; doi:10.3390/healthcare9101373)

healthcare

MDPI

Examination of electrolyte replacements in the ICU (MIMICIII) demonstrates significant waste and superfluous replacement patterns resulting in obscure clinical benefit

Supplementary Materials

Mousa Ghannam [1], Parasteh Malihi [2], Krzysztof Laudanski[3]

[1] School of Dental Medicine, Leonard Davis Institute in Healthcare Economics, University of Pennsylvania

[2] School of Dental Medicine, Leonard Davis Institute in Healthcare Economics, University of Pennsylvania

[3] Department of Anesthesiology and Critical Care, Department of Neurology, Leonard Davis Institute in Healthcare Economics; University of Pennsylvania;

Supplemental Table S1. List of exclusion ICD-9 diagnoses and their codes.

ID	N	ICD codes
Renal	4973	189.0-997.72, E879.1, V10.52-V59.4
Atrial fibrillation	2954	427.31
Acute Kidney Injury	2606	584.5-669.34
Congestive heart failure	2272	398.91-428.9
Coronary Artery Disease	919	411.1- 972.4, E942.4, V45.81
Packed Red Blood Cells	457	
Chronic Kidney Failure	319	585.1-585.4
End Stage Renal Disease	299	585.5-6, V45.1, V45.11, V56
Paralysis	125	94.1-780.72
Rhabdomyolysis	114	728.88
Dialysis	78	V45.1-V56.0
Parathyroid Disease	42	194.1-252.9
Sarcoidosis	33	135, 321.4
Pediatrics	16	N/A
Burn	9	941.0-949.0, E890.3-E988.1
Nutritional Deficiency	6	

Supplemental Table S2 Diagnoses and frequencies of the instances when over-repletion occurred.

Electrolyte	Diagnosis	N	%
Potassium	Subarachnoid Hemorrhage	3	11.12
	Skull base fracture- coma	2	7.41
	Coronary Atherosclerosis of Native Vessel	2	7.41
	DMI ketoacidosis, uncontrolled	2	7.41
	Intracerebral hemorrhage	2	7.41
	Acute myocardial infarction, inferior wall	1	3.71
	Anaerobic septicemia	1	3.71
	Aortic valve disorder	1	3.71
	Bacteremia	1	3.71
	Central Cord Syndrome; c5-7	1	3.71
Magnesium	Aortic Valve Disorder	4	7.27
	Coronary Atherosclerosis of Native Vessel	3	5.50
	Mitral Valve Disorder	3	5.50
	Anoxic Brain Damage	2	3.63
	Asthma, unspecified, w acute exacerbation	2	3.63
	Intracerebral Hemorrhage	2	3.63
	Septicemia, unspecified	2	3.63
	Acute Alcohol Intoxication	1	1.82
	Acute Alcohol Hepatitis	1	1.82
	Acute Respiratory Failure	1	1.82

Supplemental Table S3 Diagnoses and frequencies of the instances when under-repletion occurred.

Electrolyte	Diagnosis	N	%
Potassium	Subarachnoid Hemorrhage	3076	13.82
	MSRA	1464	6.58
	Acute Pancreatitis	1202	5.40
	Intracerebral Hemorrhage	1046	4.70
	Poisoning by aromatic analgesics, NEC	771	3.46
	Alcohol cirrhosis liver	743	3.34
	Septicemia, unspecified	698	3.13
	Acute respiratory failure	670	3.01
	Aplastic anemias NEC	474	2.13
	Human Immunodeficiency Virus	445	2.00
Magnesium	Pneumococcal septicemia	497	10.64
	Septicemia, unspecified	306	6.55
	Aplastic anemias, NEC	219	4.69
	Acute pancreatitis	199	4.26
	Fracture, mandible body, NEC	193	4.13
	Human immunodeficiency virus	193	4.13
	Hematemesis	108	2.31
	Pseudomonas septicemia	100	2.14
	Reaction to vascular device, implant, graft	79	1.69
	Pseudomonal pneumonia	78	1.67
Phosphate	Subarachnoid hemorrhage	51	6.89
	Septicemia, unspecified	41	5.54
	Subdural hemorrhage-coma, unspecified	38	5.14
	Other postop infection	37	5.00
	Intracerebral hemorrhage	31	4.19
	Acute respiratory failure	26	3.51
	Poisoning aromatic analgesics, NEC	26	3.51
	Acute & Chronic resp fail	24	3.24
	Acute pancreatitis	24	3.24
	DMII ketoacidosis, uncontrolled	19	2.57

Supplemental Figure S1 Top ten diagnoses when repletion was ordered when the preceding lab value was above or below the reference for potassium and magnesium.

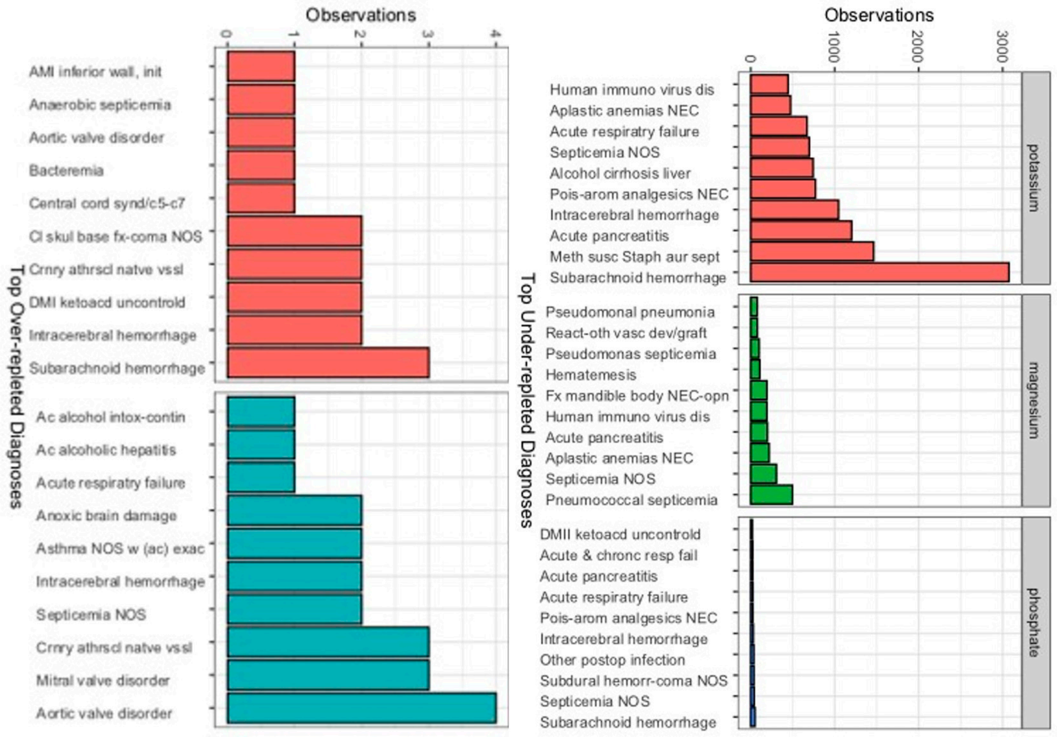

Supplement: Supplementary file 1 [file healthcare-09-01373-s001.zip › healthcare-1386346-supplementary.pdf]
